# Tracheal reconstruction with a free vascularized myofascial flap: preclinical investigation in a porcine model to human clinical application

**DOI:** 10.1038/s41598-017-10733-z

**Published:** 2017-08-30

**Authors:** Won Shik Kim, Jae Won Chang, Woo Soon Jang, Young Joon Seo, Mi-Lan Kang, Hak-Joon Sung, Da Hee Kim, Jung Min Kim, Jae Hong Park, Myung Jin Ban, Gina Na, Seung Ho Shin, Hyung Kwon Byeon, Yoon Woo Koh, Se-Heon Kim, Hong Koo Baik, Eun Chang Choi

**Affiliations:** 1 Department of Otorhinolaryngology, Yonsei University College of Medicine, Seoul, Korea; 2 Department of Otolaryngology-Head and Neck Surgery, Cancer Research Institute, Research Institute for Medical Sciences, Chungnam National University College of Medicine, Daejeon, Korea; 3 Department of Materials Science & Engineering, Yonsei University, Seoul, Korea; 4 Department of Otorhinolaryngology, Yonsei University Wonju College of Medicine, Wonju, Korea; 5 Severance Biomedical Science Institute, College of Medicine, Yonsei University, Seoul, Korea; 6 The George W. Woodruff School of Mechanical Engineering, Georgia Institute of Technology, Atlanta, United States; 7 Department of Otorhinolaryngology, Soonchunhyang University College of Medicine, Cheonan, Korea

## Abstract

Although there are various methods for tracheal reconstruction, such as a simple approximation with suturing and coverage with adjacent soft tissue or muscle, large defects >50% of the tracheal length still present a clinical challenge. Tissue engineering, a recent promising way to possibly resolve this problem, requires a long preparatory period for stem cell seeding on a scaffold and relatively invasive procedures for stem cell harvesting. As an alternative, we used a vascularized myofascial flap for tracheal reconstruction. In four porcine models, the deep inferior epigastric perforator (DIEP) was used in two and the superior epigastric artery perforator (SEAP) in two. Transformation of the surface of the transplanted myofascial flap was analyzed in the airway environment. The flaps failed in the DIEP group due to venous congestion. At 12 weeks postoperatively, none of SEAP group showed any signs of respiratory distress; the inner surface of the implant exhibited stratified squamous epithelium with sparse cilia. In the clinical setting, a patient who underwent a tracheal reconstruction with a vascularized myofascial flap and 2-year follow-up was in good health with no respiratory distress symptoms.

## Introduction

The management of tracheal defects has long been a challenge. Defects of the trachea can develop in the management of tracheal stenosis after a tracheostomy or endotracheal intubation; moreover, malignancy may develop in the vicinity of the trachea or in the trachea itself. If the defect is small, an end-to-end anastomosis can be performed after segmental resection of the stenotic portion. However, an end-to-end anastomosis is impossible for defects >50% of the total tracheal length. Thus, alternative methods for the management of long tracheal defects have been studied for many years. In 1979, Rose *et al*. performed the first reported allogeneic tracheal transplant in humans^1^. However, this method requires a suitable donor and life-long immunosuppressive agents. Methods using autologous tissues, including the esophagus and aortic grafts as a tracheal substitute, were also studied^2–5^. However, these methods have major disadvantages that include harvest-related morbidity and donor site complications. Thus, alternative methods using foreign materials or tissue engineering technology have been pursued. In 2005, Delaere *et al*. reported their management for restenosis following anastomosis after segmental resection of the trachea^6^. They used a buccal mucosal-lined fascial flap for a partial tracheal defect that developed after a longitudinal incision of the trachea for widening the tracheal lumen diameter. For vascularization of the neo-trachea, a radial forearm free flap was used. However, this procedure required temporary tracheal stenting with a silicone stent. In 2008, Macchiarini *et al*. reported the successful clinical transplantation of a tissue-engineered airway, which was an assembly of MHC-deprived donor trachea, the recipient’s autologous epithelial cells, and the recipient’s autologous chondrocytes^7^. However, the patient underwent intermittent bronchoscopic interventions and required repeated endoluminal stenting for progressive cicatricial stenosis, according to the 5-year follow-up results^8^. Their method also required long preparatory work, including the search for a suitable donor, harvesting of autologous cells, and assembling these materials using a bioreactor.

In this study, we investigated the feasibility of tracheal *reconstruction with a vascularized myofascial flap, using a deep* inferior epigastric perforator (DIEP) or superior epigastric artery perforator (SEAP) flap in a preclinical porcine model with a partial tracheal defect. We previously provided a preliminary report of a human case in which we applied the technique using a vascularized muscle fascia from an anterolateral thigh (ALT) flap to reconstruct a partial tracheal defect that developed during the surgical management of papillary thyroid cancer (PTC) invading the trachea^9^. Here, we also report 2-year follow-up data for that patient. This study establishes a theoretical foundation for the feasibility of tracheal reconstruction using a vascularized myofascial flap.

## Results

### Free vascularized myofascial flap model for the reconstruction of a tracheal defect in a porcine model

**Figure 1:**
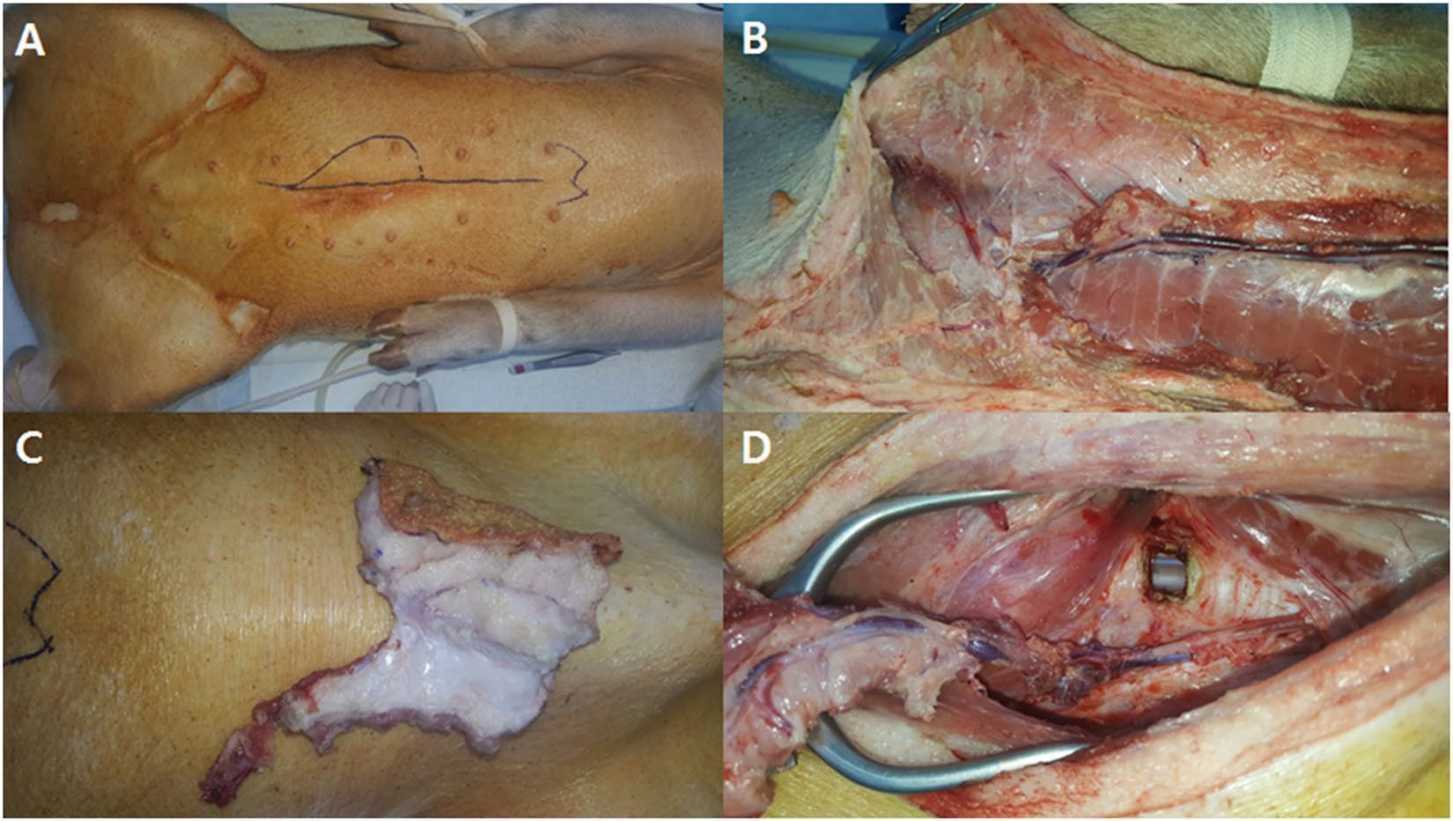
Free vascularized myofascial flap for reconstruction of a tracheal defect in a porcine model. (**A**) An elliptical vertical skin paddle was designed for the elevation of the SEAP flap on the upper abdomen. (**B**) Intramuscular dissection of the perforators from the superior epigastric vessels was performed. (**C**) The harvested flap contained the skin, subcutaneous tissue, muscle with fascia, and the superior epigastric artery with venae comitantes. (**D**) Microvascular anastomosis was performed on the previously identified carotid artery and internal jugular vein. SEAP: superior epigastric artery perforator.

We performed tracheal reconstructions using a DIEP flap in two pigs and a SEAP flap in two pigs (Table 1). The sizes of tracheal defects in these animals ranged from 54 to 72 mm^2^, and the skin paddle sizes of the flap from 32 to 40 cm^2^ (Fig. 1). Animals were euthanized at 12 weeks after the initial operation, with the exception of one pig in the DIEP group who expired on postoperative day 4 for an unidentified reason. This pig showed flap failure due to venous congestion before expiry. A second pig in the DIEP group showed flap failure also due to venous congestion and was euthanized on postoperative day 7. Thus, use of the DIEP flap was apparently not feasible in this study and in two other cases not included in the study. The cause of flap failure was venous outflow obstruction in all cases. One pig in the SEAP flap group showed donor site dehiscence, which was managed with frequent dressing changes. The flaps in the SEAP group survived, and the general condition of all animals in this group was normal until the planned euthanasia at postoperative day 84.

**Table 1:** Tracheal reconstruction results with a free vascularized myofascial flap model

Pig No.	Flap type	Postoperative complication	Follow-up (days)	Tracheal defect size (mm^2^)	Skin paddle size of flap (cm^2^)	Outcome
1	DIEP flap	Flap failure due to venous congestion	4	72	32	Expired at POD 4 due to the unidentified cause
2	DIEP flap	Flap failure due to venous congestion	7	63	40	Euthanized at POD 7 due to flap failure
3	SEAP flap	Donor site dehiscence	84	70	35	Planned euthanized at POD 84
4	SEAP flap	None	84	54	40	Planned euthanized at POD 84

DIEP: deep inferior epigastric perforator; SEAP: superior epigastric artery perforator, POD: postoperative day.

### Bronchoscopic evaluation

**Figure 2:**
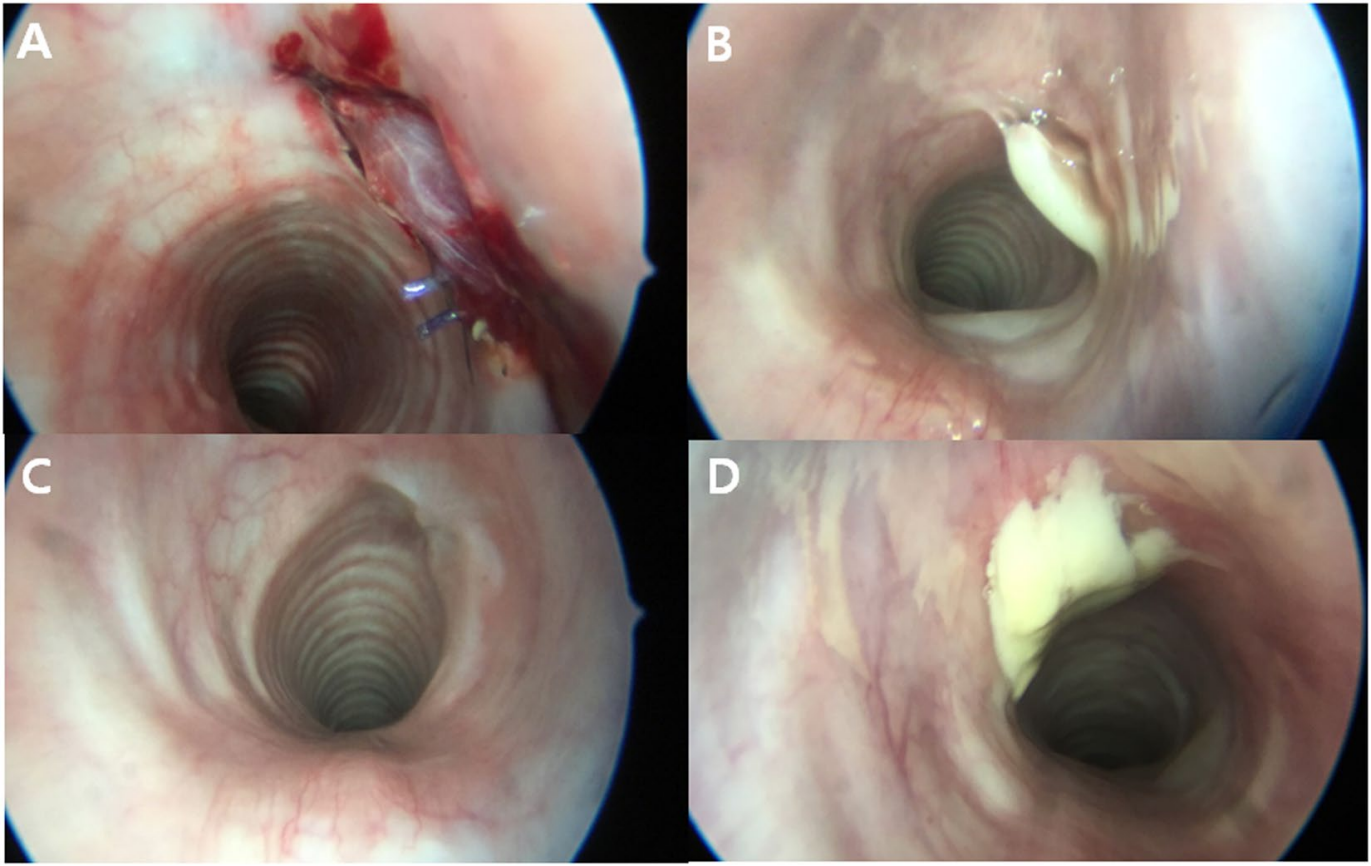
Bronchoscopic findings of a pig trachea reconstructed with SEAP and DIEP flap. (**A**) Immediate after the operation, bronchoscopy revealed pinkish muscle fascia sutured to the tracheal defect. (**B**) The yellowish secretion was noted around the reconstructed tracheal defect of SEAP flap at postoperative day 7. (**C**) The smooth intraluminal lining was noted without stenosis or granulation in SEAP group at postoperative day 84. (**D**) DIEP group which dropped out from the study, showed much more yellowish secretion than SEAP group. However, tracheal lumen compromise was not observed in the DIEP group, either. DIEP, deep inferior epigastric perforator, SEAP, superior epigastric artery perforator.

Immediately after the operation, bronchoscopy revealed a pinkish muscle fascia sutured to the tracheal defect (Fig. 2A). There were some suture knots located inside the trachea. The pinkish color of muscle fascia indicated successful restoration of blood circulation of the myofascial flap, translocated into the cervical area. SEAP group showed a yellowish secretion around the reconstructed tracheal defect at postoperative day 7 (Fig. 2B). However, there were no sign of respiratory distress throughout the entire experimental period. A smooth intraluminal lining was observed, without stenosis or granulation, at postoperative day 84 (Fig. 2C). As shown in Fig. 2D, DIEP group which dropped out from the study, showed much more yellowish secretion than SEAP group. However, tracheal lumen compromise was not observed in the DIEP group, either.

### Serial changes in exteriorized monitoring skin paddle

**Figure 3:**
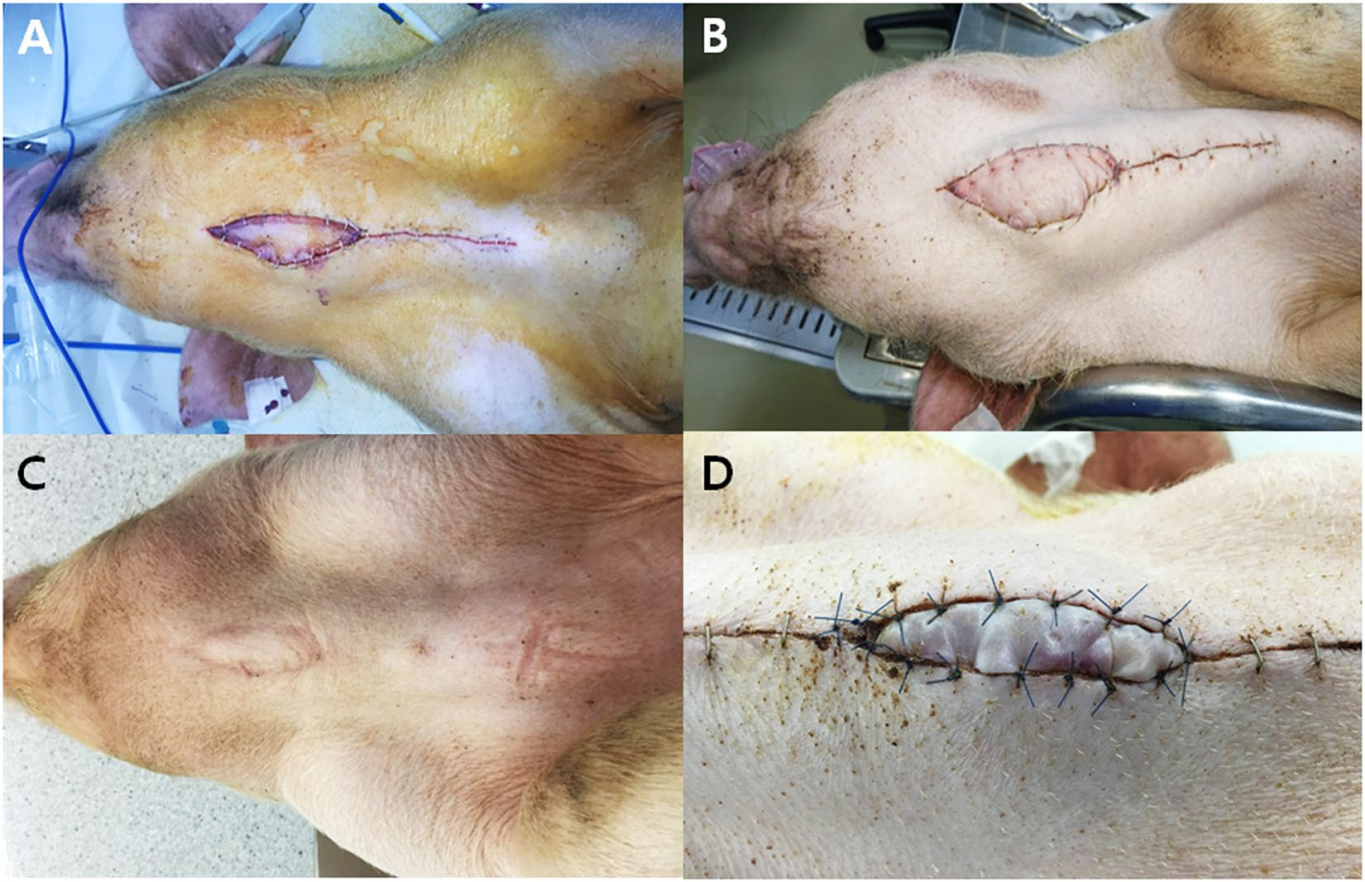
Serial change of exteriorized monitoring skin paddle of the SEAP and DIEP flap. (**A**) Immediate after the operation, the monitoring skin paddle showed pinkish color. (**B**) The monitoring skin paddle was intact and swelling in the anterior midline neck was noted at postoperative day 7. (**C**) The color of the monitoring skin paddle was similar with the surrounding neck skin and the swelling disappeared at postoperative day 84. (**D**) The color of the monitoring skin paddle changed dark indicating venous congestion. DIEP, deep inferior epigastric perforator, SEAP, superior epigastric artery perforator.

Immediately after the operation, the monitoring skin paddle of the SEAP flap showed a pinkish coloration (Fig. 3A). The monitoring skin paddle was intact, and swelling in the anterior midline neck was observed at postoperative day 7 (Fig. 3B). The color of the monitoring skin paddle was similar to that of the surrounding neck skin and the swelling disappeared by postoperative day 84 (Fig. 3C). A gradual decrease in swelling of the anterior midline neck occurred over the experimental period. The nipple contained in the skin paddle of the SEAP flap was present in the anterior neck until euthanasia. As shown in Fig. 3D, skin paddle of the DIEP group changed dark due to venous change and dropped out following study.

### Histological evaluation

**Figure 4:**
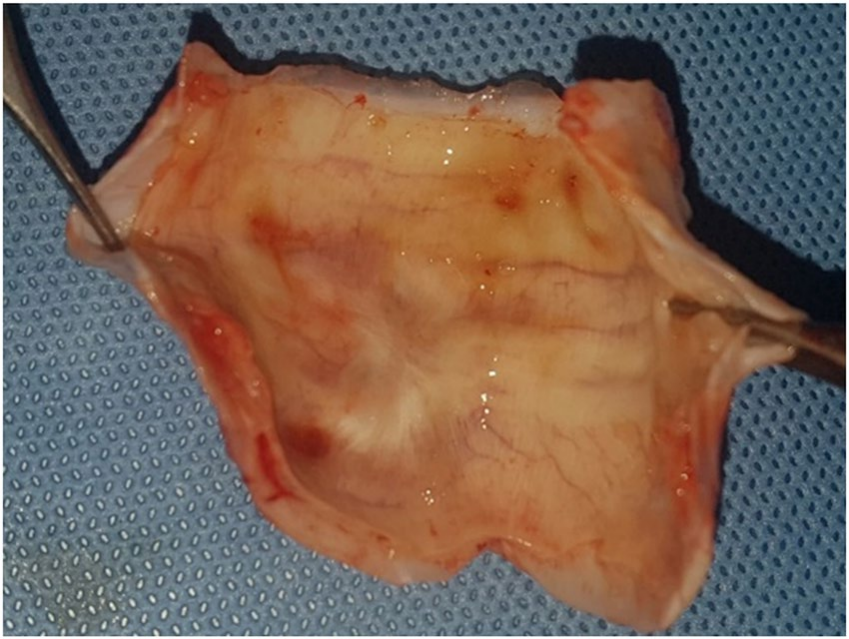
Gross morphology of a harvested specimen from pig no. 4. The intraluminal surface of the neo-trachea was smooth, with no granulation.

A tracheal specimen was harvested at postoperative day 84, together with the overlying myofascial flap, and a vertical incision was made to visualize the lumen (Fig. 4). The intraluminal surface of the neo-trachea was smooth, with no granulation.

**Figure 5:**
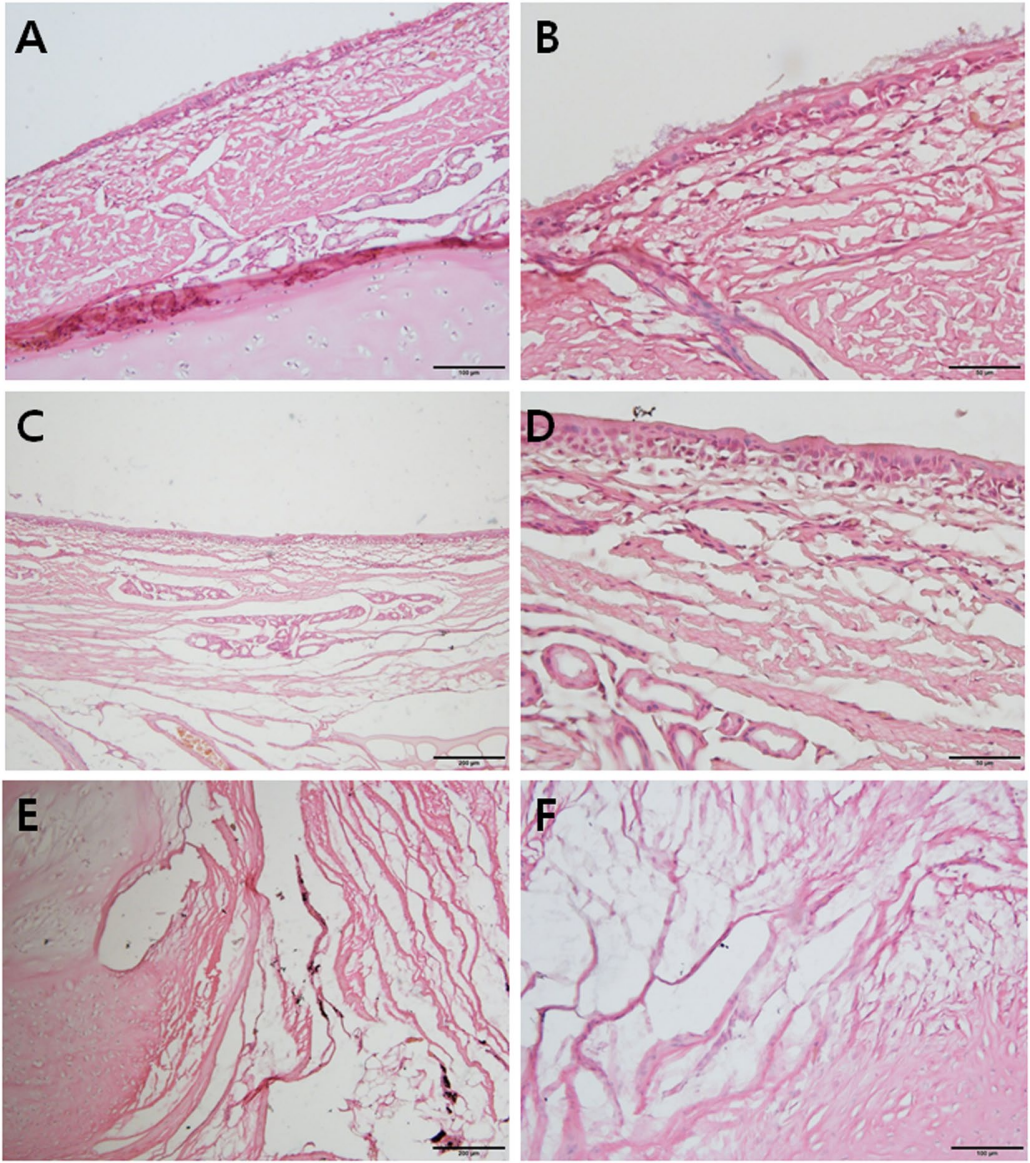
Hematoxylin and eosin staining of the trachea of pig no. 4, harvested 84 days after the surgery. (**A**) Mucociliary epithelium was noted on the fibrous connective tissue in the normal tracheal lumen. (**B**) Mucociliary epithelium in the normal tracheal lumen was revealed at high magnification (×400). Pseudostratified columnar epithelium with cilia was evident. (**C**) Epithelial cells with scanty cilia were noted on the muscular fiber tissue in the neo-tracheal lumen. (**D)** The surface of the neo-trachea was evaluated at high magnification (×400). Stratified squamous epithelium without cilia was observed. (**E**) A transition zone between the normal trachea and the neo-trachea was evident. The cartilaginous tissue (left portion) was faced with muscular fibrous tissue (right portion). (**F**) A transition zone between the muscular fibrous tissues of the neo-trachea and the cartilaginous tissue of the normal trachea was noted at high magnification (×200).

Light microscopic findings were evaluated after hematoxylin and eosin (H&E) staining. Mucociliary epithelium was noted on the fibrous connective tissue in the normal tracheal lumen (Fig. 5A). At higher magnification (×400), pseudostratified columnar epithelium with cilia was observed (Fig. 5B). Epithelial cells with scanty cilia were present in the muscular fiber tissue in the neo-tracheal lumen (Fig. 5C). The surface of the neo-trachea was evaluated at high magnification (×400), and stratified squamous epithelium without cilia was evident (Fig. 5D). A transition zone between the normal trachea and the neo-trachea was observed (Fig. 5E and F) and the cartilaginous tissue was faced with a muscular fibrous tissue. A transition zone between the muscular fibrous tissues of the neo-trachea and the cartilaginous tissue of the normal trachea was revealed at high magnification (×200; Fig. 5F) indicating histologic niche of reconstructed defect of trachea (cartilaginous tissue) through free vascularized flap (muscular fiber).

### Scanning electron microscopy

**Figure 6:**
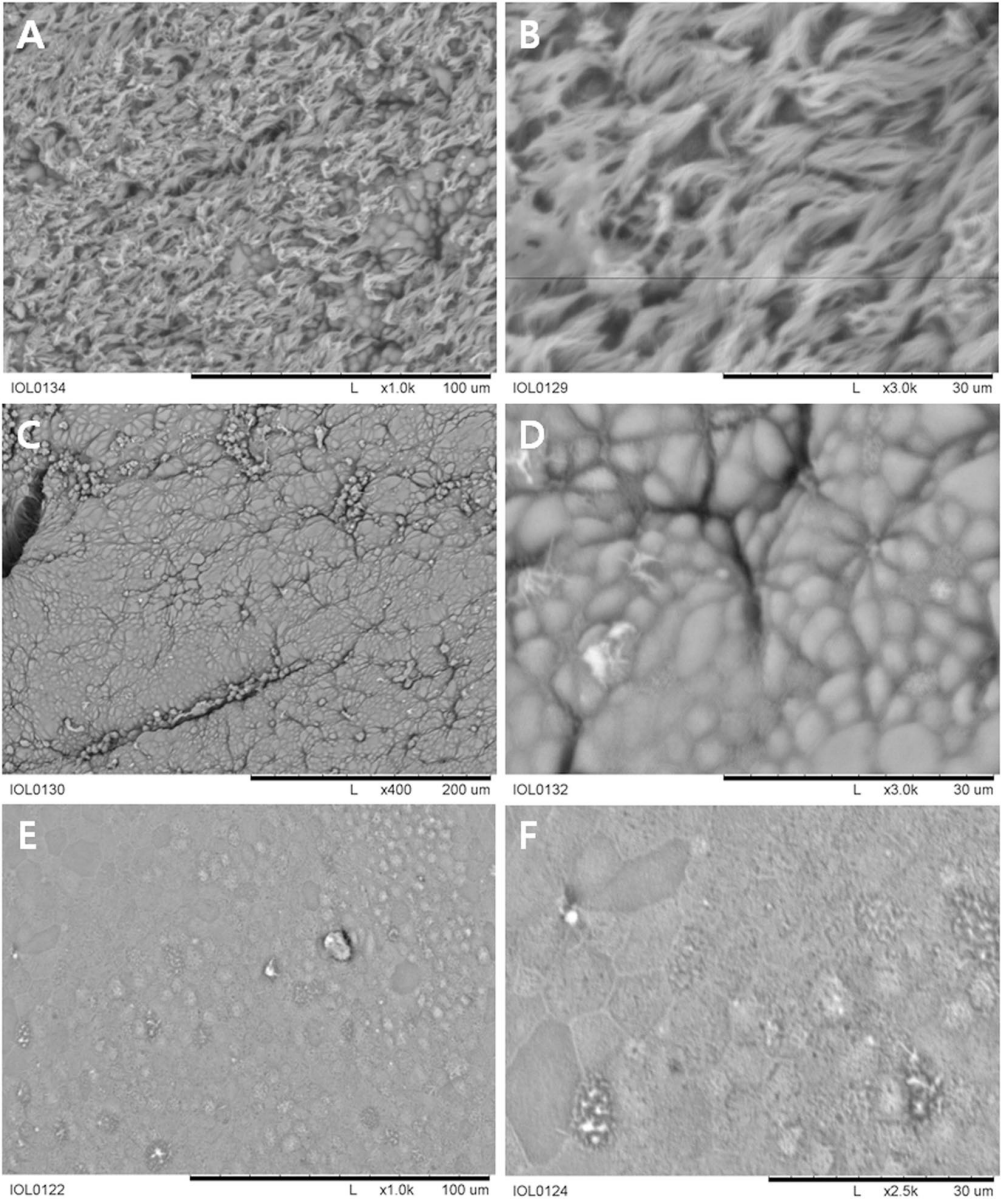
Scanning electron microscopy showing the epithelium of the trachea. (**A**) The surface of the normal trachea was crowded with many cilia. (**B**) A high-magnification image of the surface of the normal trachea revealed relatively healthy ciliated epithelium. (**C**) The surface of the neo-trachea near the transition zone between the normal trachea and the neo-trachea showed scanty cilia. (**D)** A high-magnification image of the neo-trachea surface revealed a relatively firm epithelial ultrastructure; however, ciliary structure was notably sparse. (**E**) In a small area of the neo-trachea, a dysmorphic surface was observed. (**F**) A magnified view of the dysmorphic area showed no ciliary structure.

Scanning electron microscopy (SEM) analysis was performed on the harvested trachea. The surface of the normal trachea was crowded with many cilia (Fig. 6A). A high-magnification image of the surface of the normal trachea revealed relatively healthy ciliated epithelium (Fig. 6B). The surface of the neo-trachea near the transition zone, between the normal trachea and the neo-trachea, revealed scanty cilia (Fig. 6C). A high-magnification image of the surface of the neo-trachea revealed a relatively firm epithelial ultrastructure (Fig. 6D). However, a sparse ciliary structure was also observed. In a small area of the neo-trachea, a dysmorphic surface was noted (Fig. 6E). A magnified view of this dysmorphic area showed no ciliary structure (Fig. 6F).

### Transmission electron microscopy

**Figure 7:**
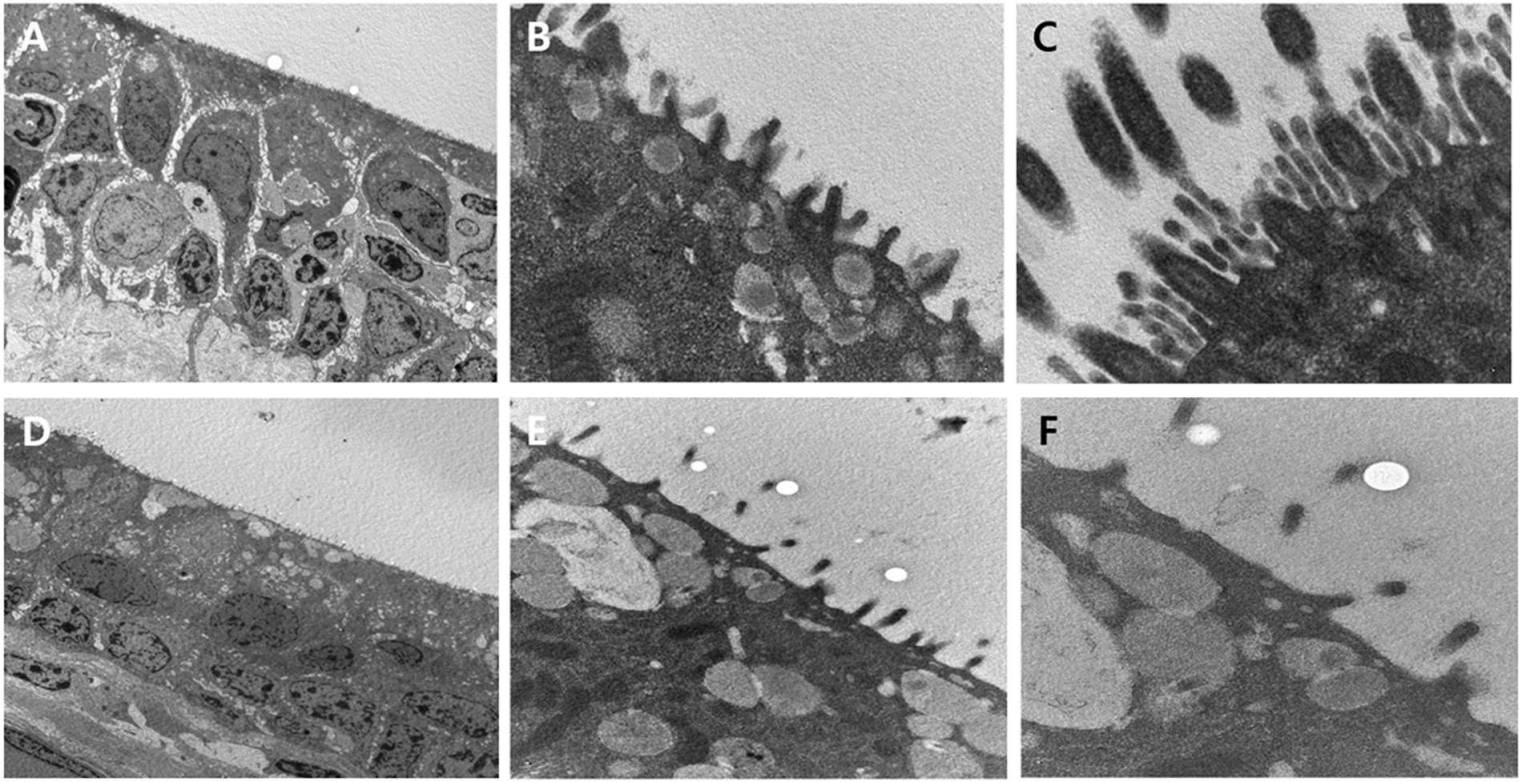
Transmission electron microscopy showing the epithelium of the trachea. (**A**) The normal trachea revealed pseudostratified columnar ciliated epithelium. (**B**) Higher magnification of the normal trachea showed cilia. (**C**) Higher magnification of the normal trachea clearly revealed the ciliary structure. (**D**) The neo-trachea showed stratified squamous epithelium with scanty cilia. (**E**) Higher magnification of the neo-trachea indicated scanty but undoubtedly present cilia. (**F**) Higher magnification of the neo-trachea revealed ciliary structures.

Transmission electron microscopy (TEM) was performed to evaluate a vertical section image of the normal trachea and the neo-trachea. The normal trachea revealed pseudostratified, columnar ciliated epithelium (Fig. 7A). A high-magnification image of the normal trachea showed cilia (Figs. 7B,C). The neo-trachea revealed stratified squamous epithelium with scanty cilia (Fig. 7D). High-magnification images of the neo-trachea also showed scanty but clearly present cilia (Fig. 7E,F).

### Human tracheal reconstruction case

**Figure 8:**
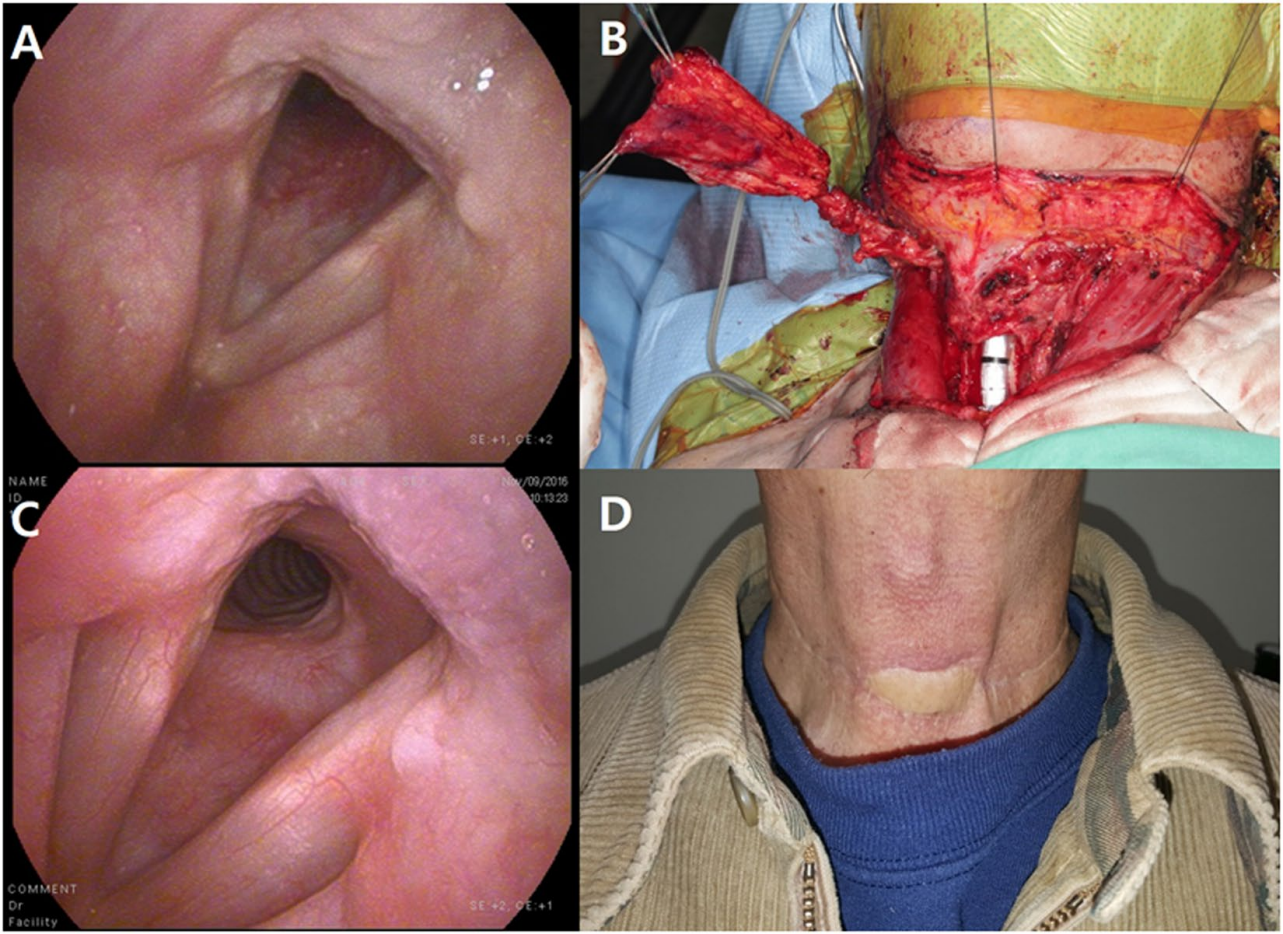
Human tracheal reconstruction case. (**A**) Preoperative flexible endoscopy revealed tracheal mucosal invasion by thyroid gland cancer. (**B**) The tracheal defect that developed after resection of the thyroid gland cancer was reconstructed using a vascularized myofascial flap from the ALT. (**C**) Postoperative flexible endoscopy showed no granulation or stenosis 24 months postoperatively. (**D**) The exteriorized skin paddle of the ALT was taken between the anterior cervical incision. ALT: anterolateral thigh.

Thyroid cancer had invaded the tracheal mucosa of our patient, primarily on the left side (Fig. 8A). After tumor removal, the tracheal defect was reconstructed using a vascularized myofascial flap from the ALT (Fig. 8B). At postoperative month 24, the patient did well without no discomfort in tracheobronchial toilet or breathing difficulties and flexible endoscopy revealed no evidence of recurrence, tracheal stenosis, or tracheal granulation (Fig. 8C). The exteriorized skin paddle of the ALT was taken between the anterior cervical incision (Fig. 8D).

## Discussion

There have been tremendous efforts to manage tracheal defects, by many methods and technologies^10, 11^. Among the suggested methods, a tracheal allograft requires a timely donor and a lifetime of immunosuppressants, limiting its common application in the management of tracheal defects caused by malignancies^12^. An autograft, such as an esophagus or aortic graft, can be a good alternative, in terms of biocompatibility; however, donor site morbidity is not negligible^4–6^. Tracheal reconstruction using artificial materials has shown unpredictable results^10^. There is a continuing unmet need in the area of the tracheal reconstruction for long segmental defects that cannot be managed with end-to-end anastomosis^13^. One of the current concepts in the management of such long segmental defects is the assembly of a biocompatible scaffold and patient-derived stem cells^10, 14^. Recently, there have been significant advances in tissue engineering and the reconstruction of tracheal defects. Many researchers in the field of tissue engineering have used cell seeding on biocompatible scaffolds, with promising results^10^. However, the shortcomings of this method include the stem cell-harvesting procedure, stem cell seeding on the scaffold using a bioreactor, and the consequent delay in timely reconstruction^10, 13^.

Delaere *et al*. successfully reconstructed tracheal defects by applying a vascularized fascia and buccal mucosa; their idea that a mucosalized fascia could be a solid substitute for a tracheal luminal surface was the foundation of our study^6^. Additionally, our group reported a successful case of tracheal defect reconstruction that developed during the surgical management of thyroid cancer using a vascularized myofascial flap^9^. The description of the histological changes in the translocated fascia directly contacting the air was the primary endpoint of this study. The long-term result of our clinical experience in tracheal reconstruction using a vascularized myofascial flap was a secondary endpoint. We expected the mucosal transformation of the myofascial surface in coping with the air passage and were hopeful that ciliary regeneration would occur from the edge between the transplanted fascia and the surrounding normal mucosa of the trachea. According to the histological results with the porcine model, a transformation to epithelial mucosa was noted, although ciliary regeneration was not achieved fully. Nevertheless, in the selected area of the neo-trachea, ciliary structures were undoubtedly observed and confirmed in immunohistochemistry using cilia specific marker (Supplementary Fig. S1). Certainly, we believe that most of these respiratory epithelial cells migrated from the adjacent normal tracheal mucosa on to the implanted tissue to form functional respiratory epithelium. However, given the presence of stem cells in fascia^15^ and the fact that the respiratory epithelium can be induced in stem cells^16^, we can extrapolate that partial regeneration of the respiratory epithelium in our data may be originated from the stem cells of the transplanted fascial system. In addition, considering the relatively short experimental time period (12 weeks), this result can reasonably be extrapolated to the complete transformation of the transplanted mucosa to airway-compatible structures. The results from both the animal model and the human case are consistent with that extrapolation. The animal model of the SEAP flap showed no sign of airway distress and generally healthy conditions until the planned euthanasia. In the human case, the patient continues to breathe without difficulty; there have been no issues, to date, in terms of bronchial toilet as of the last follow-up 25 months postoperatively.

A limitation of this study is the small defect size in the animal model. Clinically, this small tracheal defect could be managed readily with coverage, using adjacent muscle and soft tissues. We attempted to determine the transformation of the surface of the ectopically transplanted myofascial surface in the environment of the airway and the bronchial secretion passage. Thus, the findings of this study provide a foundation for future application of the vascularized myofascial flap in the reconstruction of a circumferential tracheal defect.

In addition, it is difficult to make a reliable comparison because of the small sample size of each group, although it was due to the unexpected flap failure of DIEP group. However, despite the small sample size of this study, we provide a proof-of-concept for the feasibility of free flap grafts for use in tracheal reconstruction. The possible cause of our unexpected failure of DIEP group may be because the diameter of pedicles (1.1~1.3 mm for artery, 0.6~0.7 mm for vena comitans) in DIEP group were smaller than that of SEAP group (1.7~2.2 mm for artery, 1.8~2.4 mm for vena comitans). This caused technological difficulties in venous anastomosis, resulting in venous outflow obstruction leading to flap failure.

Another weakness of the study is the obscurity of the transformation mechanism of the transplanted myofascial flap surface. Histological analysis showed that stratified squamous epithelium and dysmorphic epithelium existed simultaneously on the transplanted surface. The transformation could have started from the edge of the myofascial flap, with the influence of the adjacent normal tracheal mucosa, or could be the result of environmental stimuli, including high-pressure air passage through the transplanted myofascial surface. We could not further study the serial timeline changes due to small numbers of animals and the small size of the defect, which limited the ability to take serial tracheal biopsies. Follow-up studies should focus on the transformation mechanism of the ectopically transplanted myofascial flap.

In conclusion, tracheal reconstruction with a free vascularized myofascial flap was shown to be feasible in this study. The surface of the transplanted myofascial flap transformed into epithelium with sparse cilia. There was no tracheal granulation or stenosis in both an animal model and in clinical application. These results can provide a foundation for the application of free vascularized myofascial flaps in the management of long segmental tracheal defects. Follow-up studies are necessary to determine the transformation mechanism of the transplanted myofascial flap in the airway.

## Methods

### Animal model and surgical procedure

Four female Yorkshire pigs, weighing 25–30 kg, were obtained from XP Bio, Seoul, Korea. The study was approved by the Department of Laboratory Animal Resources, Yonsei Biomedical Research Institute, Yonsei University College of Medicine, which is accredited by the Association for Assessment and Accreditation of Laboratory Animal Care International. We followed the guidelines for the Care and Use of Laboratory Animals of the Institute of Laboratory Animal Resources Commission on Life Sciences National Research Council, USA.

All pigs were acclimated for 1 week before the operation. Before general anesthesia, the pigs fasted for over 12 h. Anesthesia was started with an intramuscular injection of the mixture of alfaxan 1 mg/kg, xylazine 2 mg/kg, and azaperone 2 mg/kg. After securing an intravenous (IV) route, ketorolac 1 mg/kg was injected via the IV line. Atropine 0.04 mg/kg and cefazolin 30 mg/kg were administered intramuscularly. After the anterior neck and the abdomen of the pig were shaved, intubation with an orotracheal tube was performed. The anesthesia was maintained with isoflurane 2%. With a vertical midline incision in the anterior neck, the strap muscles were dissected and retracted laterally. The trachea was exposed from the first ring to the thoracic inlet. The common carotid artery and the internal jugular vein were exposed and prepared for the anastomosis. The window was made in the 2nd or 3rd tracheal cartilage, ~1 cm in width. The endotracheal tube was still in place, bypassing the tracheal defect, and aeration through the tube was continued using the ventilator. The DIEP or the SEAP flap harvest was performed, according to the method described by Bodin *et al*.^17^. For the DIEP flap, an elliptical vertical skin paddle was incised on the inferior abdomen. The skin flap was elevated from the rectus abdominis muscle sheath with preservation of the perforators to the skin flap. Small inferior epigastric vessels were identified and preserved. For the SEAP flap, a longitudinal elliptical skin paddle was designed on the upper abdomen (Fig. 1A). After flap elevation, intramuscular dissection of the perforators from the superior epigastric vessels was performed (Fig. 1B). The harvested flap contained the skin, the subcutaneous tissue, the muscle with fascia, and the superior epigastric artery with venae comitantes (Fig. 1C). A microvascular anastomosis was performed to the previously identified carotid artery and the internal jugular vein (Fig. 1D). Then, the tracheal window was covered and sutured with the muscle fascia of the DIEP or the SEAP flap using Vicryl (Ethicon, Germany) 3-0. A skin paddle of the DIEP or the SEAP flap was exteriorized and sutured to the cervical midline skin incision. The abdominal skin incision was closed. After finishing the surgical procedure, the pigs received peroral amoxicillin-clavulanate 14 mg/kg and meloxicam 0.2 mg/kg, as antibiotics and pain management, respectively, for 1 week.

### Bronchoscopic evaluation

A smart phone-endoscopy system, consisting of a smart phone (iPhone model 6 s, Apple Inc., CA, USA), an endoscope adaptor for a smart phone (MobileOptx, MobileOptx, PA, USA), and a rigid endoscope (Karl Storz Hopkins II 26006AA, Karl Storz GmbH & Co. KG, Tuttlingen, Germany), was used for bronchoscopic evaluations. Serial changes in the inner lining of the translocated muscular fascia in the neo-trachea were evaluated twice per week with the smart phone-endoscopy system.

### Histological evaluation

The specimen of interest was intended to be harvested at 12 weeks after the initial operation. The transplanted neo-trachea was harvested *en bloc* with the adjacent normal tracheal mucosa under general anesthesia. After harvesting the specimen, the animal was euthanized with an intravenous injection of potassium chloride. For hematoxylin and eosin (H&E) staining, the specimens were placed in 10% neutral formalin solution. After fixation, the specimens were embedded in paraffin wax and sectioned (section thickness: 4 µm). Specimens were stained with H&E and examined under light microscopy. In addition, the frozen sections were analyzed by immunohistochemistry of β-tubulin for cilia. For the immunohistochemistry, the tracheal airway in pigs of each group were embedded in Tissue-Tek O.C.T. compound (Sakura Finetek, Torrance, CA, USA). A rabbit anti-β tubulin monoclonal antibody (Abcam, Cambridge, UK; 1/100) was used as primary antibody and treated at 4 °C for O/N. Secondary and thirdly antibodies were biotinylated goat anti-rabbit IgG (Vector, Burlingame, CA, USA; 1/200) and Texas Red^®^ streptavidin (Vector; 1/50), respectively. The antibodies were diluted in 1% bovine serum albumin and incubated in RT for 1 h. Immunofluorescence analysis was performed by using a Leica DMi8 fluorescence microscope (Leica, Wetzlar, Germany). For the processing and analyses of images, LAS AF Lite software (Leica) was used.

### Scanning electron microscopy

For scanning electron microscopy (SEM) analysis, the specimens were fixed in phosphate-buffered saline containing 2% glutaraldehyde and 0.1% paraformaldehyde for 2 h. Then, they were stained with 1% OsO4. Specimens were coated with gold using an EiKO IB-3 ion sputter coater (EiKO, Shawnee, KS, USA) and examined with a field emission gun (FEG) scanning electron microscope (S-800, Hitachi, Japan) at an acceleration voltage of 20 kV. Images were processed with ESCAN 4000 software (Bummi Universe Co., Ltd., Seoul, Korea). For cell length measurements, more than 100 straight-lined cells were chosen randomly from digitized SEM images, and the distance between the two poles of each cell was automatically calculated.

### Transmission electron microscopy

Transmission electron microscopy (TEM) was performed to evaluate the transformation of the vascularized myofascial flap in air- and bronchial secretion-contacting environments. The specimens were fixed in Karnovsky solution (2% glutaraldehyde and 2% paraformaldehyde), rinsed in 0.1 M sodium cacodylate, and post-fixed with Lee’s fixative (a 1:1:1 mix of 0.5% RuO4, 2% OsO4, and 0.2 M cacodylate buffer) at room temperature for 90 min. This procedure was designed to minimize ciliary injury and to facilitate viewing of the ciliated cells. The trachea was also decalcified by immersion in Calci-Clear Rapid (National Diagnostics) for 24 h. Then, we cut cross-sections of the trachea in a plane containing the normal trachea, the neo-trachea, and the transition zone. Next, each section was dehydrated in an alcohol solution substituted with propylene oxide, and embedded in the Epon mixture. The embedded sections were double-stained with uranyl acetate and lead citrate. The sections were then analyzed by TEM (JEM-1200 EDXII microscope, 80 kV; JEOL, Tokyo, Japan).

### Human tracheal reconstruction case

We previously described a human case report in which a tracheal defect caused during the surgical management of PTC invading the trachea was reconstructed with a free vascularized ALT myofascial flap^9^. Briefly, a 70-year-old man with a cytology-confirmed PTC invading the tracheal cartilage underwent a total thyroidectomy, central compartment neck dissection, tracheal window resection of the invading trachea (defect size, 35 × 20 mm, from 1^st^ to 3^rd^ tracheal ring, more than anterior half), and tracheal reconstruction with an ALT myofascial flap. The fascial side of the ALT was sutured to the tracheal defect, and a small elliptical part of the skin paddle of the ALT was exteriorized between the upper and lower neck skin flaps. Extubation of the endotracheal tube at 12 h after the operation was performed uneventfully. The patient was discharged without severe complications on postoperative day 18. High-dose (150 mCi) radioiodine ablation was performed at 5 months postoperatively. This study was approved by the Institutional Review Board of Yonsei University College of Medicine, and the requirement to obtain informed consent was waived.

## Electronic supplementary material

Supplementary figure S1
