# Supplementary material for: Tracheal reconstruction with a free vascularized myofascial flap: preclinical investigation in a porcine model to human clinical application
Source: Sci Rep. 2017 Aug 30;7:10022. doi: 10.1038/s41598-017-10733-z (PMC5577286; doi:10.1038/s41598-017-10733-z)
Supplement: Supplementary file 1 — Supplementary figure S1 [file 41598_2017_10733_MOESM1_ESM.pdf]

Supplementary Information

Tracheal reconstruction with a free vascularized myofascial flap: preclinical investigation in a porcine model to human clinical application

Won Shik Kim[a], Jae Won Chang[b], Woo Soon Jang[c], Young Joon Seo[d], Mi-Lan Kang[e], Hak-Joon Sung[e,f], Da Hee Kim[a], Jung Min Kim[a], Jae Hong Park[g], Myung Jin Ban[g], Gina Na[a], Seung Ho Shin[a], Hyung Kwon Byeon[a], Yoon Woo Koh[a], Se-Heon Kim[a], Hong Koo Baik[c], Eun Chang Choi[a]

[a]Department of Otorhinolaryngology, Yonsei University College of Medicine, Seoul, Korea

[b]Department of Otolaryngology-Head and Neck Surgery, Cancer Research Institute, Research Institute for Medical Sciences, Chungnam National University College of Medicine, Daejeon, Korea

[c]Department of Materials Science & Engineering, Yonsei University, Seoul, Korea

[d]Department of Otorhinolaryngology, Yonsei University Wonju College of Medicine, Wonju, Korea

[e]Severance Biomedical Science Institute, College of Medicine, Yonsei University, Seoul, Korea

[f]The George W. Woodruff School of Mechanical Engineering, Georgia Institute of Technology, Atlanta, United States

[g]Department of Otorhinolaryngology, Soonchunhyang University College of Medicine, Cheonan, Korea

Correspondence and requests for materials should be addressed to W.S.K. and J.W.C

(wskim78@yuhs.ac & strive1005@hanmail.net)

Supplementary Figure S1

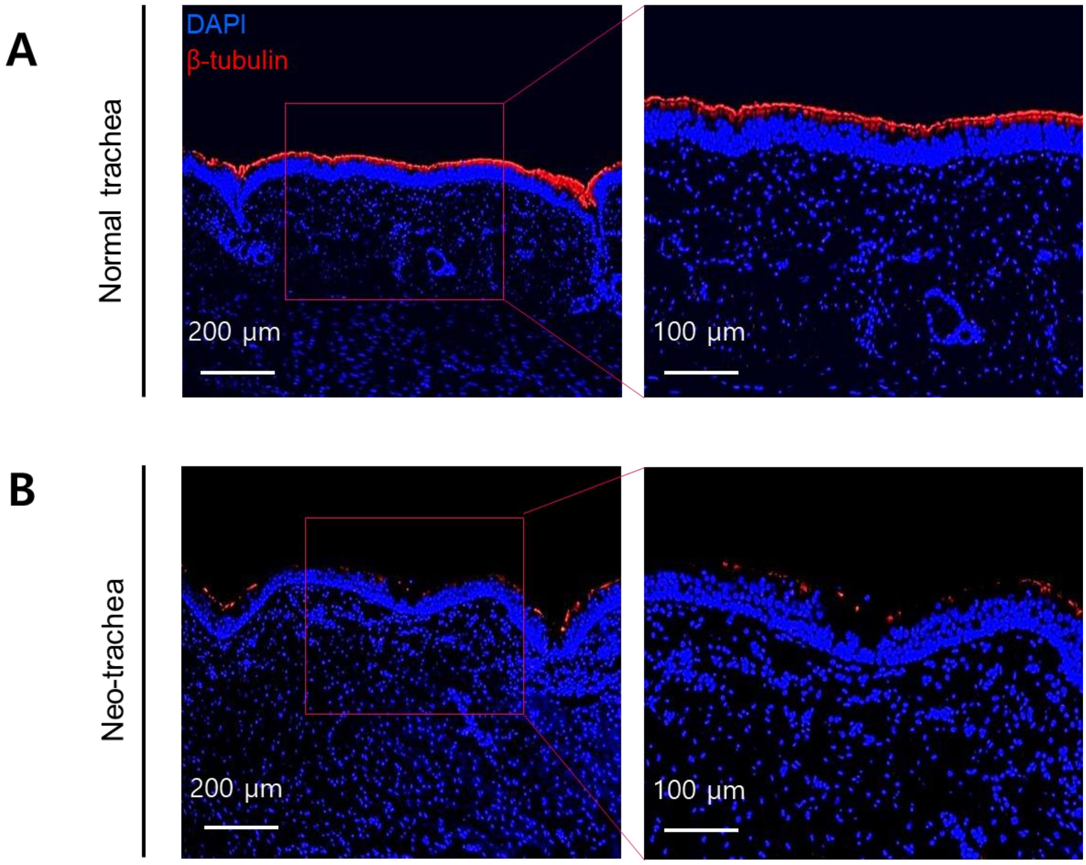

Figure S1. ***In vitro* evaluation of fluorescence-labeled cilia in normal trachea and neo-tracheal epithelium at post-operative 12 weeks.** A. The normal trachea revealed fluorescence-labeled cilia. Higher magnification of the normal trachea showed cilia lining whole epithelium. B. The neo-trachea showed scanty fluorescence-labeled cilia. Higher magnification of the neo-trachea indicated scanty but obviously present cilia.
